# Associations between Mental Health and Oral Health among Korean Adolescents: Analysis of the National Surveys (2008–2017)

**DOI:** 10.3390/ijerph182010660

**Published:** 2021-10-12

**Authors:** Kyeong-Hee Lee, Won-Kee Lee, Eun-Seo Jung, Yoon-Young Choi

**Affiliations:** 1 Department of Dental Hygiene, College of Bioecological Health, Shinhan University, Uijeongbu 11644, Korea; khlee@shinhan.ac.kr (K.-H.L.); dentalmien@hanmail.net (E.-S.J.); 2 Department of Medical Informatics, School of Medicine, KyungPook National University, Daegu 41944, Korea; wonlee@knu.ac.kr

**Keywords:** adolescents, mental health, national survey, oral health, suicide attempt, suicide ideation

## Abstract

We aimed to analyze the trends and associations between the oral and mental health of Korean adolescents using the Korea Youth Risk Behavior Web-based Surveys. In total, 713,713 adolescents who participated in the surveys were included. To examine trends in oral and mental health, Pearson’s correlation coefficients were calculated between the weighted percentages and years, and the regression line slope was assessed to estimate annual changes. A complex sample logistic regression analysis was also performed. Experiencing oral symptoms and suicide-related factors both showed a gradually decreasing trend from 2008 and slight increases in 2017 and 2016, respectively. Compared with adolescents who had no oral symptoms, those with all six oral symptoms showed an increase in suicide attempts in the last 12 months, suicide ideation, and feelings of sadness and despair. In conclusion, experiencing more oral symptoms is associated with an increased risk of suicide-related factors.

## 1. Introduction

Health in adolescence is extremely important since it sets the foundation for health in adulthood [1]. The perceptions and attitudes towards health that are formed during adolescence are closely related to the quality of life [2]. However, as a transition period from childhood to adulthood, adolescence is a time of rapid biological, social, and cognitive change while the individual is still psychologically immature. Accordingly, excessive stress in adolescence can have negative effects on both mental and physical health [3], and this can be easily overlooked in healthcare [4]. Adolescence is a developmental stage in which maintaining a healthy mental state is challenging. Adolescence is also characterized by impulsive tendencies, which can lead to suicide [5]. In Korea, adolescent suicide is becoming a social issue, with adolescents often failing to recognize their self-worth and the importance of health as a result of stress-inducing factors such as sudden social changes, academic-focused education, and fiercely competitive preparation for university admission [6]. Oral healthcare is very important for adolescents as they often experience various oral diseases, such as stomatitis, dental caries, and periodontal disease [5]. According to the 2018 Korean Children’s Oral Health Survey, more than half of the 12-year-old-participants had experienced dental caries in a permanent tooth [7], and more than one-third of the people who were receiving treatment for dental caries were children or adolescents [8]. Even though these oral diseases have negative effects on both school life and daily living, oral health remains the most neglected aspect of adolescent healthcare [9,10].

The mental and oral health of adolescents has been reported to be related [11,12]. Poor oral health can affect eating and speech, which also adversely affects other social and psychological areas [13]. Patients with mental illness are likely to develop oral diseases for reasons such as difficulties in oral hygiene management and taking medicine [14,15]. DeFalco et al. [3] found that oral health in adolescents is associated with health behaviors, such as alcohol consumption and smoking, mental health factors such as stress, and socioeconomic factors such as parents’ educational achievements and economic status. Freeman [16] observed that adolescent oral health behaviors are strongly affected by psychological and social backgrounds. Schüler et al. [17] conducted a study of children and adolescents aged 6–16 years and reported that those with mental disability and psycho-emotional disorders had worse oral health, more caries, and more odontogenic infections. Research has also shown that the rate of toothbrushing is higher among adolescents who have no stress or suicidal ideation [18]. However, although there have been many previous studies on the effects of mental health on oral health, most focused on depression and perceived stress as characteristics of adolescent mental health. Additionally, only a few studies examined changes in adolescent oral and mental health by analyzing long-term trends [19,20]. A study in Lebanon reported that suicidal ideation and suicide planning significantly decreased between 2005 and 2017; however, the prevalence of inadequate oral hygiene remained as high as 40% throughout the survey year [19]. In a study in the Philippines that analyzed health risk behaviors data for 2003, 2007, and 2011, suicidal ideation decreased in boys, but increased in girls; both boys and girls showed a trend of improving oral hygiene management behaviors [20].

This study aimed to analyze trends in the oral and mental health of adolescents over the last 10 years using data from the 4th–13th Korea Youth Risk Behavior Web-based Surveys (KYRBS; 2008–2017), which are national surveys of Korean adolescents, and to investigate the relationships between the oral and psychiatric health statuses of the adolescents. The null hypotheses of this study were as follows: (1) The oral and mental health of Korean adolescents did not change from 2008 to 2017, and (2) oral and mental health of Korean adolescents are not related. 

## 2. Materials & Methods

### 2.1. Research Data and Participants

This epidemiological study involved the analysis of secondary data extracted from the KYRBS. The KYRBS are anonymous online surveys of adolescents that were designated as a source of government-approved statistical data based on the South Korean National Health Promotion Act. KYRBS is a nationwide cross-sectional survey that focuses on health-risk behaviors among middle- and high-school students. The questionnaire consists of 128 items in 14 areas on the health status, including mental and oral health. To obtain a representative sample of Korean adolescents, KYRBS uses a multi-stage cluster sampling design. Schools are used as the primary sampling units within each cluster in the first stage of the sampling. The classes for each grade in the selected sample school are used as the secondary extraction units, and all students in the sample class are considered the subjects of the survey [21,22]. The sample population of this study was middle and high school students in Korea who participated in the 4th–13th KYRBS (2008–2017). Of the total 739,850 sample population, 96.5% participated in these surveys (*n* = 713,713). 

### 2.2. Variables

Sex and age were used as demographic variables. The socioeconomic variables included city size, school type, and economic status. City size was categorized as ‘major city’, ‘small/medium city’, or ‘county’; school type was categorized as ‘middle school’, ‘general high school’, or ‘specialized high school’; and economic status was categorized as ‘upper’, ‘middle-upper’, ‘middle’, ‘middle-lower’, or ‘lower’. 

Oral health was measured using the six oral symptoms highlighted by the World Health Organization [23]. The oral symptoms investigated were tooth fracture, pain while eating, shooting or throbbing pain, gum pain and bleeding, pain in the tongue or the inside of the cheek, and bad breath. Participants reported whether they had experienced any of these symptoms in the last 12 months (Supplemental Table S1). Mental health was measured by investigating whether participants had experienced suicidal ideation, a suicide attempt, or feelings of sadness and despair in the last year (Supplemental Table S2).

### 2.3. Data Analyses

The frequency of experience of oral symptoms was calculated and categorized according to the survey year and sociodemographic characteristics, and the weighted percentages were calculated by applying the sampling weights. To examine the trends in oral health over the last 10 years, Pearson’s correlation coefficients were obtained between the years and the weighted percentages of oral health symptoms in each year. To estimate the mean annual change in oral health symptoms, the slope of the regression line was calculated. To examine the extent of changes in the mental health of adolescents, the odds ratio (OR) and 95% confidence interval (CI) for each year were calculated relative to those of 2008. To examine the trends of the mean changes over the 10-year period, the year, which was the independent variable, was converted into a continuous variable and the ORs and 95% CIs were calculated. To analyze the association between oral health and mental health, a simple logistic regression was performed, with the total number of oral health symptoms experienced as the independent variable. A multiple logistic regression analysis was performed to adjust sex, age, economic status, and survey year. Because feelings of sadness and despair can be a risk factor of suicidal behavior, this factor was included as an additional confounding variable in the multiple regression models using suicide attempt and suicidal ideation as dependent variables. These ORs and 95% CIs were obtained using complex samples logistic regression. SAS 9.4 (Copyright (c) 2002–2012 by SAS Institute Inc., Cary, NC, USA) was used for statistical analysis. The statistical significance level was set at α = 0.05.

## 3. Results

### 3.1. Sociodemographic Characteristics of the Participants

Participants were evenly distributed by year, with a percentage of around 10.0% for each year. Of the participants, 52.5% were male, and 47.5% were female. Regarding age, the most common age was 16 years (17.4%), whereas 12 years was the least common age (7.2%) (Supplemental Table S3).

### 3.2. Oral Health

When the participants’ experience of oral symptoms was examined according to survey year, the most commonly experienced symptom was dental pain when eating (38.2% on average), whereas the least commonly experienced symptom was a pain in the tongue or the inside of the cheek (12.6%). Participants experienced 1.36 symptoms on average (Table 1). 

The correlation coefficient for the survey year was negative for all oral symptoms, showing a lower prevalence of symptoms over time. Of all six symptoms, ‘dental pain when eating’ showed the strongest correlation with survey year, with a sample correlation coefficient of −0.98 (*p* < 0.001); the symptom with the weakest correlation was ‘bad breath’, with a sample correlation coefficient of −0.86 (*p* = 0.001). A decreasing trend was noted in the annual changes of all oral symptoms. ‘Shooting or throbbing pain’ decreased the most, reducing from 30.9% in 2008 to 23.6% in 2017 (s = −0.935; *p* < 0.001). The mean number of oral symptoms decreased by an average of 0.039 per year (*p* < 0.001), reducing from 1.57 in 2008 to 1.21 in 2016, and increasing slightly to 1.24 in 2017.

### 3.3. Mental Health

All three mental health characteristics showed annually decreasing trends (Table 2). Compared with 2008, the frequency of ‘suicide attempt in the last 12 months’ decreased in every year except 2010 (OR, 1.06; CI, 0.99–1.14); the characteristic also had an OR of 0.49 in 2016 and 0.54 in 2017, compared with 2008. When the 10-year trend for suicide attempts was analyzed, the OR was 0.916, representing a mean decrease of 8.4% year-on-year. Compared with 2008, the frequency of ‘suicidal ideation in the last 12 months’ increased every year up to 2011, but decreased from 2012; the OR was 0.59 in 2017, representing a 1.69-fold decrease in the number of participants who experienced suicide ideation compared with the number recorded in 2008. The mean annual decrease in suicidal ideation was 7.3%. The frequency of ‘feelings of sadness and despair in the last 12 months’ decreased continuously from 2008; the OR was 0.53 in 2017, representing a 1.89-fold decrease in the number of participants who experienced feelings of sadness and despair compared with the number recorded in 2008. The mean annual decrease in feelings of sadness and despair was 8.2%.

In terms of the absolute decrease in the frequency of the three mental health characteristics, ‘suicide attempts’ decreased by 0.31% per year, reducing from 4.7% in 2008 to 2.6% in 2017; ‘suicide ideation’ decreased by 1.03% per year; and ‘feelings of sadness and despair’ decreased by 1.78% per year.

### 3.4. Relationship between Oral Symptoms and Mental Health

According to simple logistic regression models, we observed a trend of worse mental health with an increasing number of oral symptoms experienced (Table 3). Compared with adolescents who experienced no oral symptoms, those who experienced all six symptoms showed a 6.19-fold higher risk of ‘suicide attempts’, a 3.81-fold higher risk of ‘suicidal ideation’, and a 3.00-fold higher risk of ‘feelings of sadness and despair’; all the results were statistically significant (*p* < 0.001). Even after controlling for all general characteristics and other confounding variables, compared with adolescents who experienced no oral symptoms, those who experienced all six symptoms showed a 3.38-fold higher risk of ‘suicide attempts’, a 2.40-fold higher risk of ‘suicidal ideation’, and a 2.63-fold higher risk of ‘feelings of sadness and despair’, all of which were also statistically significant (*p* < 0.001). 

Worse mental health was noted among female students, students who felt sadness, and students who reported better economic statuses. Older age was associated with reduced suicide attempts and suicidal ideation, but with increased feelings of sadness and despair. Even after controlling for the number of oral symptoms experienced and other factors, suicide attempts, suicidal ideation, and feelings of sadness and despair decreased significantly over time (*p* < 0.001).

## 4. Discussion

Establishing strategies based on the understanding of the health characteristics of adolescents is essential in creating an environment where adolescents can maintain their physical health and be psychologically happy. However, amidst the rapid pace of change in modern society, it is impossible to understand adolescent health characteristics by only assessing a specific time point. Hence, we analyzed trends in the oral and mental health of Korean adolescents over a period of 10 years and investigated the correlations between the oral and mental health of the participants. According to the findings, both null hypotheses were rejected in this study.

We observed that the number of oral symptoms showed a decreasing trend over time. The symptoms that showed the largest decrease over time were ‘dental pain when eating’ and ‘shooting or throbbing pain’. We surmised that these symptoms could be related to dental caries [24]. This result is similar to that of a study by Arantes et al. [25], in which trends in the oral health statuses of Brazilian adolescents were analyzed. In that study, the mean decayed, missing, or filled tooth surfaces index showed a decreasing trend over time, reducing from 4.95 in 2004 to 2.39 in 2007. Fluoride and dental sealants are applied to prevent the development of dental caries [26]; dental sealants are especially effective in preventing the development of caries on the occlusal surface. From December 2009, the South Korean national health insurance for children aged 6–14 years included preventive treatment, which involved the application of a dental sealant on the first molars. From May 2013, the preventive treatment was expanded to include treatment of the first and second molars for all children under 18 years old [27]. The occurrence of dental caries-related oral symptoms in adolescents seems to have gradually decreased as a result of these proactive prevention policies.

Along with dental caries, the major disease that affects the oral health of adolescents is a periodontal disease that causes several symptoms such as ‘Gum pain and bleeding’, ‘Pain in the tongue or the inside of the cheek’, and ‘Bad breath’ [28]. Although many risk factors, such as age, smoking, alcohol consumption, and systemic disease, are related to periodontal disease [29], an accumulation of biofilm is the main cause of periodontal disease [30]. Therefore, good oral hygiene is essential to prevent periodontal disease, and the decrease in periodontal symptoms in our study may be due to continuous oral health education. Of the six oral symptoms, ‘tooth fracture’ also showed a decreasing trend, which can be attributed to school safety education [31]. Continuous and improved school safety education including content regarding the use of smart devices, vehicle safety, and wearing of mouth guards should be provided.

Despite the trend of a decreasing number of oral symptoms, even in 2017, which was the last survey year included in the present study, adolescents experienced an average of 1.36 of the six oral symptoms, a slight increase compared to the previous year. Alcohol consumption and smoking rates of Korean adolescents showed a steady decline in the 2000s but increased again in 2017 [32]. Because drinking and smoking are strong risk factors for oral disease, an increase in alcohol consumption and smoking may be the cause of the increase in the number of oral symptoms in the present study. Therefore, further preventive measures and politics need to be applied through the continual provision of adequate oral healthcare.

Adolescent suicide is an incident that exists on a continuum and usually starts with suicidal ideation [33]. Eighty percent of all adolescents who attempted suicide were reported to have previously expressed suicide-related intent [34]. Since adolescent suicide is impulsive and interventions are impossible after the incident, it is essential to predict suicide risk factors and implement preventive interventions rather than retrospective strategies. In the present study, suicide attempts, suicidal ideation, and feelings of sadness and despair, which were examined as mental health characteristics, showed a gradually decreasing trend from 2008 to 2015. This finding is contrary to that of a study of 10–19-year-old adolescents in the United States between 1975 and 2016 [35], which showed a continuous increase in suicide rate from 2007 to 2016. Another study, in which the mortality database of the World Health Organization was used to investigate the suicide rate among 15–19-year-old adolescents in South Korea, Japan, the United States, and Finland [36], indicated that Korean adolescents showed the lowest suicide rate. In 2011, the South Korean government established the Basic Plan for Suicide Prevention in an effort to prevent adolescent suicide and has been establishing linkages between schools and local mental health-related services [37]. In 2012, the Ministry of Education implemented proactive policies to prevent student suicide and has focused on detecting high-risk groups and strengthening intervention systems. Various mental health-related services are being provided by the Ministry of Health and Welfare and other relevant departments [38]. This suggests that the decrease in suicide-related indices among Korean adolescents is related to the efforts of the Korean government. However, given that suicide has been the leading cause of death among Korean youths (9–24 years) since 2008 [39], this problem should not be taken lightly.

Upon analyzing the relationship between experiencing oral symptoms and mental health, we found that the risk of having mental health characteristics increased with an increasing number of oral symptoms experienced. Similarly, Chun et al. [12] reported that people who brushed their teeth less often and experienced more oral symptoms showed higher perceived stress. Kim & Kim [40] reported that academic stress, which is known to be a major risk factor for adolescent suicidal ideation, is also indirectly related to oral health-improving behaviors. Several mechanisms have been suggested for the relationship between oral and mental health. Patients with depression can suffer from poor oral health, due to poor oral hygiene caused by self-neglect and xerostomia caused by the use of antidepressants [14]. In patients with bipolar affective disorder, overzealous brushing or flossing during the manic phase can cause tooth wear and mucosal or gingival lacerations [15]. In addition, lithium use is associated with xerostomia and stomatitis [15]. Because normal oral function is a fundamental factor in a person’s quality of life, poor oral health also can undermine mental health [13]. Poor oral health can cause problems with both speech and eating, which can exacerbate low self-esteem, social withdrawal, and isolation. In addition, oral diseases are associated with several systemic diseases, which may cause secondary mental illness [41,42].

This study has several limitations. Firstly, since the adolescents’ experiences of oral symptoms and their mental health characteristics were measured subjectively through a health questionnaire rather than by clinical diagnoses by certified physicians, the exact presence or absence of disease could not be determined. Additionally, we cannot exclude the possibility of recall bias, which is common in questionnaire surveys. Secondly, since the variables were limited to those selected by the KYRBS, we could not account for potential confounding variables such as oral health behavior, dental visits, dietary habits, systemic disease, and family history. Moreover, since this was a cross-sectional study, we could not readily infer causality for the association between oral and mental health. Despite these limitations, this study has considerable value because we used reliable and representative data at a national scale to investigate oral and mental health among Korean adolescents, and identified patterns of change over time by analyzing trends.

## 5. Conclusions

The frequency of experiencing oral symptoms and suicide-related factors showed a gradually decreasing trend from 2008 but began to slightly increase again around 2016 and 2017. Adolescents who experienced more oral symptoms were at a higher risk of suicidal ideation and suicide attempts. This demonstrates a relationship between adolescent oral health and mental health. In the future, it will be beneficial to perform a systematic analysis using diagnoses by certified physicians based on clinical tests. Additionally, future bidirectional research on oral health and mental health in adolescents will be valuable, as it can clearly elucidate the causal relationships between oral health and mental health.

## Figures and Tables

**Table 1:** Participants’ experience of oral symptoms according to the survey year.

Year	*n*	Tooth Fracture *	Dental Pain When Eating *	Shooting or Throbbing Pain *	Gum Pain and Bleeding *	Pain in the Tongue or the Inside of the Cheek *	Bad Breath *	Number of Oral Symptoms Experienced ^†^
2008	75,238	16.9	41.1	30.9	26.7	14.7	26.6	1.57
2009	75,066	15.9	39.9	29.3	24.6	14.4	25.4	1.50
2010	73,238	13.7	39.1	30.3	23.4	13.4	23.2	1.43
2011	75,643	13.3	39.3	29.7	21.2	13.1	23.4	1.40
2012	74,186	12.7	38.6	28.0	19.8	12.2	23.4	1.35
2013	72,435	12.4	38.0	27.4	19.5	11.8	22.4	1.32
2014	72,060	12.2	36.7	24.7	19.9	11.5	21.3	1.26
2015	68,043	11.4	36.7	23.7	19.5	11.1	21.7	1.24
2016	65,528	11.4	35.3	23.6	18.5	11.2	21.5	1.21
2017	62,276	11.2	35.8	23.6	19.5	11.7	22.3	1.24
Average		13.2	38.2	27.4	21.4	12.6	23.2	1.36
R ^‡^		−0.93 (<0.001)	−0.98 (<0.001)	−0.96 (<0.001)	−0.89 (0.001)	−0.92 (<0.001)	−0.86 (0.001)	−0.97 (<0.001)
S ^¶^		−0.593 (<0.001)	−0.608 (<0.001)	−0.935 (<0.001)	−0.795 (0.001)	−0.401 (<0.001)	−0.490 (0.001)	−0.039 (<0.001)

* Weighted percentages for complex sampling. ^†^ Weighted means of total oral symptoms for complex sampling. ^‡^ Pearson’s sample correlation coefficient between weighted percentages and years. ^¶^ The slope of linear regression was assessed to estimate the annual change in weighted percentages. ^‡,¶^ () is the *p*-value for each estimate.

**Table 2:** Mental health characteristics according to survey year.

Year	Suicide Attempt within the Last 12 Months		Suicidal Ideation within the Last 12 Months		Feelings of Sadness or Despair within the Last 12 Months	
	% *	OR (95% CI) ^†^	% *	OR (95% CI) ^†^	% *	OR (95% CI) ^†^
2008	4.7	1	18.9	1	38.8	1
2009	4.6	0.97 (0.90–1.04)	19.1	1.01 (0.97–1.06)	37.5	0.95 (0.91–0.98)
2010	5.0	1.06 (0.99–1.14)	19.3	1.03 (0.98–1.07)	37.4	0.94 (0.90–0.98)
2011	4.3	0.90 (0.84–0.97)	19.6	1.04 (1.00–1.09)	32.8	0.77 (0.74–0.80)
2012	4.1	0.86 (0.80–0.92)	18.3	0.96 (0.92–1.00)	30.5	0.69 (0.67–0.72)
2013	4.1	0.87 (0.81–0.93)	16.6	0.85 (0.82–0.89)	30.9	0.70 (0.68–0.73)
2014	2.9	0.61 (0.57–0.66)	13.1	0.64 (0.62–0.67)	26.7	0.57 (0.55–0.60)
2015	2.4	0.50 (0.47–0.55)	11.7	0.57 (0.54–0.59)	23.6	0.49 (0.47–0.51)
2016	2.4	0.49 (0.45–0.53)	12.1	0.59 (0.56–0.62)	25.5	0.54 (0.52–0.56)
2017	2.6	0.54 (0.50–0.58)	12.1	0.59 (0.56–0.62)	25.1	0.53 (0.51–0.55)
Average	3.8		16.3		31.3	
Change in OR year by year ^‡^		0.916 (0.911–0.922)		0.927 (0.923–0.930)		0.918 (0.915–0.923)
r ^¶^	−0.92 (<0.001)		−0.91 (<0.001)		−0.96 (<0.001)	
s ^§^	−0.31 (<0.001)		−1.03 (<0.001)		−1.78 (<0.001)	

* Weighted percentages for complex sampling. ^†^ Weighted odds ratio (OR) and 95% confidence interval (CI) for complex sampling. ^‡^ Average weighted OR and 95% CI of increasing years for complex sampling. ^¶^ Pearson’s sample correlation coefficient between weighted percentages and years. ^§^ The slope of linear regression was assessed to estimate the annual change in weighted percentages. ^¶,§^ () is the *p*-value for each estimate.

**Table 3:** Relationship between oral health and mental health.

Variable		Suicide Attempts		Suicidal Ideation		Feelings of Sadness and Despair	
		OR (95% CI) ^†^	aOR (95% CI) ^‡^	OR (95% CI) ^†^	aOR (95% CI) ^‡^	OR (95% CI) ^†^	aOR (95% CI) ^‡^
Number of oral symptoms	0	1	1	1	1	1	1
	1	1.07 (1.03–1.12) ***	0.90 (0.86–0.94) ***	1.34 (1.31–1.37) ***	1.16 (1.14–1.19) ***	1.34 (1.32–1.36) ***	1.20 (1.25–1.30) ***
	2	1.42 (1.36–1.48) ***	1.03 (0.99–1.08)	1.84 (1.80–1.88) ***	1.41 (1.37–1.44) ***	1.79 (1.76–1.82) ***	1.62 (1.59–1.65) ***
	3	1.91 (1.82–2.00) ***	1.21 (1.15–1.27) ***	2.46 (2.40–2.52) ***	1.66 (1.61–1.70) ***	2.39 (2.34–2.44) ***	2.10 (2.06–2.14) ***
	4	2.66 (2.53–2.80) ***	1.50 (1.42–1.58) ***	3.23 (3.14–3.33) ***	1.96 (1.90–2.02) ***	3.14 (3.06–3.22) ***	2.71 (2.64–2.80) ***
	5	3.28 (3.06–3.50) ***	1.73 (1.62–1.86) ***	3.94 (3.79–4.10) ***	2.28 (2.18–2.38) ***	3.70 (3.57–3.84) ***	3.23 (3.11–3.35) ***
	6	6.19 (5.72–6.71) ***	3.93 (3.60–4.29) ***	3.81 (3.60–4.04) ***	2.56 (2.40–2.73) ***	3.00 (2.84–3.16) ***	2.77 (2.63–2.92) ***
Sex	Male	1	1	1	1	1	1
	Female	1.74 (1.68–1.80) ***	1.35 (1.31–1.39) ***	1.66(1.63–1.70) ***	1.33 (1.30–1.35) ***	1.60 (1.58–1.63) ***	1.53 (1.51–1.55) ***
Sadness	No	1	1	1	1	-	
	Yes	10.93 (10.53–11.36) ***	9.90 (9.51–10.30) ***	10.27 (10.08–10.45) ***	9.16 (8.99–9.33) ***		
Age		0.91 (0.90–0.92) ***	0.86 (0.85–0.86) ***	0.99 (0.99–1.00) *	0.92 (0.92–0.93) ***	1.09 (1.08–1.09) ***	1.06 (1.06–1.07) ***
Economic status		1.27 (1.24–1.29) ***	1.14 (1.12–1.16) ***	1.27 (1.26–1.28) ***	1.15 (1.14–1.16) ***	1.22 (1.21–1.23) ***	1.12 (1.11–1.13) ***
Year		0.92 (0.91–0.92) ***	0.96 (0.96–0.97) ***	0.93 (0.92–0.93) ***	0.97 (0.97–0.97) ***	0.92 (0.91–0.92) ***	0.93 (0.92–0.93) ***

Number of oral symptoms (qualitative: 0 (None)—6), sex (qualitative: 1 [male]—2 [female]), sadness (qualitative: 1 (No)—2 (Yes)), age (quantitative: 12–18), economic status (Likert scale: 1 (bad)—5 (good)), year (quantitative: 2008–2017) ^†^ Weighted crude odds ratio (OR) and 95% confidence interval (CI) calculated using simple logistic regression for complex sampling. ^‡^ Weighted adjusted OR and 95% CI calculated using multiple logistic regression for complex sampling. * *p* < 0.05, *** *p* < 0.001.

## Data Availability

The data supporting the findings of this study are available on the KYRBS homepage (http://www.kdca.go.kr/yhs/ (accessed on 21 June 2021)).

## Supplementary Materials

The following are available online at https://www.mdpi.com/article/10.3390/ijerph182010660/s1, Table S1: Questionnaire items related to oral health; Table S2: Questionnaire items related to mental health. Table S3: General characteristics of the participants.
