# Supplementary material for: Associations between Mental Health and Oral Health among Korean Adolescents: Analysis of the National Surveys (2008–2017)
Source: Int J Environ Res Public Health. 2021 Oct 12;18(20):10660. doi: 10.3390/ijerph182010660 (PMC8535659; doi:10.3390/ijerph182010660)
Supplement: Supplementary file 1 [file ijerph-18-10660-s001.zip › ijerph-1380714-supplementary.pdf]

Supplemental Table S1. Questionnaire items related to oral health

	In the last 12 months, have you experienced the following symptoms?		
	Symptom	No	Yes
1	A tooth breaking or fracturing		
2	Tooth pain when drinking (or eating) beverages (or food) that are hot or cold		
3	Shooting or throbbing pain in the teeth		
4	Pain or bleeding of the gums		
5	Throbbing pain in the tongue or the inside of the cheek		
6	Unpleasant smell from the mouth		

Supplemental Table S2. Questionnaire items related to mental health

	Question	No	Yes
1	In the last 12 months, have you felt such sadness or despair that it interrupted your daily activities constantly for at least two weeks?		
2	In the last 12 months, have you thought seriously about suicide?		
3	In the last 12 months, have you attempted suicide?		

Supplemental Table S3. General characteristics of the participants

Characteristic	Division	Frequency*, n	Weighted†, %
Survey year	2008	75,238	10.9
	2009	75,066	11.0
	2010	73,238	10.8
	2011	75,643	10.6
	2012	74,186	10.4
	2013	72,435	10.2
	2014	72,060	9.8
	2015	68,043	9.3
	2016	65,528	8.8
	2017	62,276	8.4
Sex	Male	367,131	52.5
	Female	346,582	47.5
Age	12	55,058	7.2
	13	120,597	16.1
	14	122,613	16.7
	15	121,645	17.3
	16	120,265	17.4
	17	118,974	17.3
	18	54,561	8.0
City size	Major city	332,652	46.5
	Small/medium city	303,544	47.4
	County region	77,517	6.1
School type	Middle school	360,782	48.7
	General high school	275,490	40.6
	Specialized high school	77,441	10.7
Economic status	Upper	53,343	7.7
	Middle-upper	173,761	24.9
	Middle	337,252	47.0
	Middle-lower	115,196	15.8
	Lower	34,161	4.7
Number of oral symptoms experienced	0	258,653	36.3
	1	181,291	25.4
	2	128,994	18.1
	3	78,484	10.9
	4	40,503	5.6
	5	17,803	2.5
	6	7,985	1.1
**Total**		713,713	100.0

*Frequency of the unweighted sample.

†Weighted percentages for complex sampling.
